# Ethnicity recording in health and social care data collections in Ireland: where and how is it measured and what is it used for?

**DOI:** 10.1186/s12939-019-1107-y

**Published:** 2019-12-31

**Authors:** Ailish Hannigan, Nazmy Villarroel, Maria Roura, Joseph LeMaster, Alphonse Basogomba, Colette Bradley, Anne MacFarlane

**Affiliations:** 1 Public and Patient Involvement Research Unit, Graduate Entry Medical School, University of Limerick, Limerick, V94 T9PX Ireland; 2 Health Research Institute, University of Limerick, Limerick, Ireland; 3 Department of Family Medicine and Community Health, University of Kansas School of Medicine, Kansas City, Kansas USA; 4 Intercultural and Diversity Education Centre - Ireland (IDEC-Ireland), Ennis, Co. Clare Ireland; 5 Shannon Family Resource Centre, Shannon, Co. Clare Ireland

**Keywords:** Ethnicity, Equality monitoring, Health information systems

## Abstract

**Background:**

In the European Union (EU), discrimination based on racial and ethnic origin is prohibited under the Racial Equality Directive. Ireland is one of only three EU countries where a legal duty of equality data collection is placed on public bodies. It provides an important context in which to study ethnic equality monitoring; however no systematic mapping of where it occurs in health information systems has been carried out. The aim of this study is to identify all existing national health and social care data collections with information on ethnicity and to explore how this data has been collected and used.

**Methods:**

An electronic search of a national catalogue of health and social care data collections (*N* = 97) was carried out to identify any collections which contained information on ethnicity. Data dictionaries were searched and key informants contacted. For each of the data collections that collected information on ethnicity, data was extracted on the ethnic categories used and how this data is collected; the completeness of ethnicity recording; and other measures related to ethnicity in the data collection. Relevant outputs for these data collections, related to ethnicity, were identified through key informants and electronic searches.

**Results:**

Of the 97 data collections, 14 (14%) collected information on ethnic or cultural background. Country of birth was collected by 10 of these 14 data collections. Most used the ethnic categories in the Census and recommended that ethnicity should be self-identified and not assigned. Reported rates of identification were generally high (≥90%). Data collections which recorded ethnicity tended to be focused on potentially high-risk populations with no routine recording in primary care. There were some examples of where ethnic equality monitoring had informed targeted interventions e.g. vaccination awareness initiatives or cultural training for healthcare staff.

**Conclusions:**

Despite strong policy and legal imperatives, there is limited data collection of ethnicity in health and social care data collections in Ireland. While there are some examples of where differences by ethnicity have been identified and acted upon, a more coordinated and comprehensive approach to the collection, quality and utilization of ethnicity data is needed to promote health equity.

## Background

International policy recommends continuous, cost-effective monitoring of data related to equality and discrimination to identify any gaps that exist between groups and inform policy and targeted interventions [1]. In the European Union (EU), discrimination based on racial and ethnic origin is prohibited under the Racial Equality Directive. This directive, which was adopted in 2000, provides a framework for combating discrimination and giving effect to the principle of equal treatment [2].

The European Committee of Social Rights has identified a duty on national authorities to collect equality data; however in a review of data collection on ethnicity in the EU, Farkas reported that obligations to collect racial and ethnic data are not generally codified in law in the EU Member States [3]. Measuring racial and ethnic origin is sensitive and challenging [4, 5]. Ethnicity is a fluid, subjective and contextual concept with many dimensions including language, religion and country of origin. Its complexity can be difficult to capture in discrete categories, though Farkas concludes that if carefully applied and interpreted, the categories used can reflect its complexity [3].

Farkas reported that only three EU member states (Finland, the United Kingdom and Ireland) had placed a duty of equality data collection on public bodies. The focus of this study is on Ireland where the Irish Human Rights and Equality Commission Act 2014 (Article 42) introduced a public duty in relation to human rights and equality [6]. Public duty charges Irish publicly funded bodies to have regard to the need to eliminate discrimination, promote equality and protect human rights both in relation to staff and those to whom services are provided. It establishes requirements for an equality and human rights assessment by each organisation and an annual report on evidence of progress in furthering equality goals. Nine grounds for discrimination have been identified in Irish law including race, nationality or ethnic origin and membership of an indigenous ethnic minority, Irish Travellers.^1^ Irish Travellers have consistently lower healthy life expectancy and disability-free life expectancy than the general population in Ireland [7] and can encounter significant barriers to accessing health care [8].

Ireland is an increasingly diverse society, with the number of foreign-born residents representing 17% of the population in the most recent Census in 2016, the fifth highest rate of foreign-born in the EU Member States [9]. The majority of foreign-born are from other EU countries and reflect free movement in the EU; however, there are also strong migration flows from non-EU countries with migrants from non-EU countries representing a third of inward migration in 2018 [10]. Language barriers, lack of access to interpreters and legal issues impact on health care for some migrants in Ireland [8, 11].

**Fig. 1:**
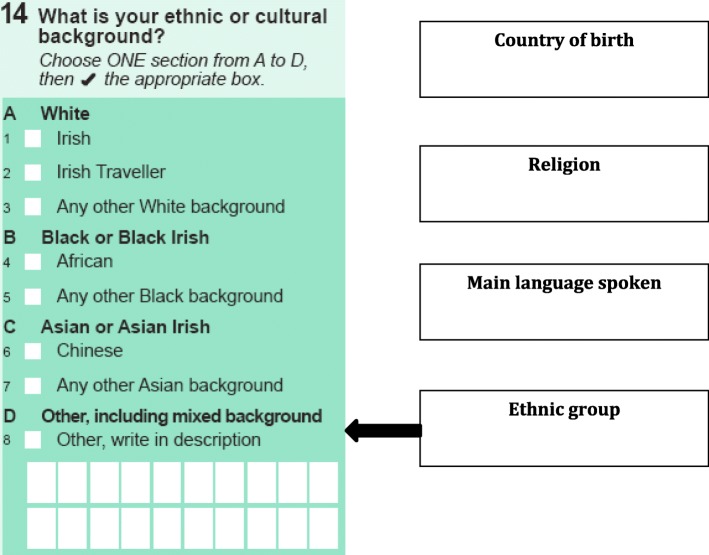
Ethnic identifier using a suite of questions including the Irish Census (2011 and 2016) question on ethnic or cultural background

In line with international policy and public duty legislation, the national public healthcare provider (the Health Service Executive, HSE) has proposed a system-level response to record data about ethnicity: an ethnic identifier (see Fig. 1) embedded in existing health information systems. The importance of data disaggregated on the basis of ethnicity has been acknowledged by the HSE as a means of addressing inequality and preventing discrimination [12]. Ethnic equality monitoring is also supported by Pavee Point, the national NGO working to promote rights of Irish Travellers, and members of the Roma community in Ireland [13]. Pavee Point is one of the few Traveller and Roma organisations in Europe that advocate for the collection of disaggregated ethnic data [3].

Given the policy and legal imperatives and the support of an advocacy organisation representing some ethnic minorities, Ireland provides an important context in which to study ethnic equality monitoring; however, no systematic mapping of where it occurs in health information systems has been carried out. This is problematic for three reasons. First, there is a lack of knowledge about the implementation of policy and legal imperatives. Second, data may be available but may not be used or fully exploited if relevant stakeholders do not know it is being collected. Third, the nature and quality of data collection has not been examined in order to identify scope for comparative analysis or changes needed to improve standardization.

As part of a larger project on building the evidence base on ethnic minority health in Ireland [14], the aim of this study is to identify all existing national health and social care data collections with information on ethnicity and to explore how this data has been collected and used.

## Methods

The Health Information and Quality Authority (HIQA) is an independent authority in the Republic of Ireland which reports to the Minister for Health. HIQA has responsibility for setting standards for all aspects of health information, monitoring compliance with those standards and evaluating the quality of the information available on health and social care. In 2010, HIQA were tasked with creating a catalogue of all national collections of routine health and social care data in the Republic of Ireland. National data collections include administrative collections, censuses, national routine surveys, and patient registries. Data collections were identified through desktop research, input from key stakeholders and previously published data reviews and inventories of data in Ireland. The third edition of the catalogue was published in 2018 and included 97 national health and social care data collections (75 national data collections, 10 national surveys, nine data collections with regional coverage, three censuses) and 23 collated sources of health and social care information including national performance and activity reports [15]. Information reported for each data collection included the managing organisation and contact details, data collection methodology, the size and geographical scope of the data collection, data content, and a link to the data dictionary where available.

We electronically searched the catalogue for any data collections that contained information on ethnicity, collected at an individual level. We also searched data dictionaries (where available) and contacted key informants for data collections.

We extracted the following information for each of the data collections that collected information on ethnicity:

- The ethnic categories used and how this data is collected, e.g. self-identified by the service user or assigned by service providers;
- The completeness of ethnicity recording;
- Other measures related to ethnicity in the data collection, e.g. language spoken at home, country of birth, citizenship, religion;
- Whether the data collection had a web link to a data dictionary or data collection form, a process for sharing data externally and whether identifiers were available in the data collection, e.g. name, address, date of birth, to support data linkage;

For data collections that collected information on ethnicity, we searched their web address for any reports of analysis of healthcare utilisation, access and/or health outcomes by ethnicity. We contacted key informants for these data collections and used a Google Scholar search with the data collection name as a search term to identify any relevant outputs (traditional academic or grey literature) related to ethnicity. We also reviewed a recently published scoping review of migrant health research in Ireland for any relevant studies [16].

A descriptive analysis of extracted data was carried out across all data collections with information on ethnicity.

## Results

Of the 97 national health and social care data collections in the catalogue, 14 (14%) collected information on ethnic or cultural background. The 14 data collections included the Census of Population, eight national data collections and five national surveys of the population. In addition to ethnic or cultural background, country of birth was included in 10 of the 14 data collections and seven collected data on length of stay in Ireland, for those not born in the country (see Tables 1 and 2). Eleven of the 14 data collections had a publically available web link to a data dictionary or data collection form and 12 of the 14 had a process for sharing data externally. Common identifiers that could be used for data linkage, e.g. name, address and date of birth, were included in the Census of Population and five of the eight national data collections (Table 1).

**Table 1:** Recording of ethnicity and other related variables in the Census and national health and social care data collections

Data collection	Web link to data dictionary/data collection form	Process for data access	Identifiers	Ethnic or cultural background	Country of birth	Citizenship	Nationality	Length of stay	Language	Religion
Census										
Census of the population 2016	✓	✓	Name, address, DOB	Census categories (see Table 2)	✓		✓	✓	✓	✓
National health and social care data collections										
National Psychiatric In-Patient Reporting system	✓	✓	Address, DOB	Census categories and Roma	✓					
National Drug Treatment Reporting System	✓	✓	Name, address, DOB, health identifier	Census categories and Roma	✓				✓	
Perinatal mortality surveillance system	✓			Census categories						
Severe Maternal Morbidity Audit	✓	✓		Census categories						
Surveillance of homebirths database		✓		Census categories						
Computerised Infectious Disease Reporting e.g. Gonorrhoea	✓	✓	Name, address, DOB	Census categories and Roma	✓					
Cystic Fibrosis Registry		✓	Name, address, DOB	Census categories						
Irish Childhood Diabetes Register			DOB	Census categories	✓			✓

**Table 2:** Recording of ethnicity and other related variables in national surveys

Data Collection	Web link to data dictionary/data collection form	Process for data access	Ethnic or cultural background	Country of birth	Citizenship	Nationality	Length of stay	Language	Religion
Growing up in Ireland National Longitudinal study of children	✓	✓	Census categories	✓	✓		✓	✓	✓
National Patient Experience Survey	✓	✓	Census categories						
Survey of Lifestyles, Attitudes and Nutrition	✓	✓	Census categories	✓			✓		
Healthy Ireland survey	✓	✓	Census categories	✓					
European Social Survey	✓	✓	Belonging to an ethnic minority group	✓	✓		✓	✓	

### National censuses

One of the three national censuses in the catalogue recorded ethnic or cultural background.

The *Census of Population* is carried out by the Central Statistics Office every five years and has recorded ethnic or cultural background since 2006. The ethnic categories used and data from the most recent Census in 2016 is given in Table 3 with ethnic or cultural background self-identified by 97.4% of the population [17]. The majority ethnic group was *White Irish* (82.2% of the population) but almost one in five of the population identified as a member of an ethnic minority. Ethnic minority groups include *Other White* (9.5% of the population) and *Irish Traveller* (0.7% of the population). Roma is currently not included as an ethnic category in the Census. The size of the Roma community in Ireland has been estimated at between four and five thousand people (0.1% of the total population) [18].

**Table 3:** Ethnic or cultural background Census of the Population 2016

Ethnic or cultural background	Total (%)
White	
Irish	3,854,226 (82.2)
Irish Traveller	30,987 (0.7)
Other White	446,727 (9.5)
Black or Black Irish	
African	57,850 (1.2)
Any other Black background	6789 (0.2)
Asian or Asian Irish	
Chinese	19,447 (0.4)
Any other Asian background	79,273 (1.7)
Other including mixed background	70,603 (1.5)
Not stated	124,019 (2.6)
Total	4,689,921

**Source:** Central Statistics Office. Census 2016 Summary Results - Part 1

Health information collected in the Census includes self-rating of health, chronic health conditions and disabilities.

### National health and social care data collections

Eight of the 75 national health and social care data collections recorded ethnic or cultural background.

The *National Drug Treatment Reporting System* (NDTRS) is an epidemiological database on treated problem drug and alcohol use in Ireland. It has a well-defined protocol for the collection of ethnicity which, in line with best practice [19], requires ethnicity to be self-identified by clients and not assigned by staff. It uses the Census categories with the addition of Roma. It includes an option if the service user ‘Does not wish to answer’ or ‘Not recorded’ if there was no opportunity to ask the question, however an analysis of cases from 2007 to 2010 reported that ethnicity was recorded for 99.4% of cases [20]. Secondary data analysis of the NDTRS has been used to provide evidence on addiction services accessed by Irish Travellers [20]. This evidence has informed the provision of culturally appropriate training for staff and peer workers to support Irish Travellers in accessing addiction services [21].

The *National Psychiatric In-Patient Reporting System* gathers data on patient admissions and discharges from psychiatric hospitals and units throughout Ireland. It uses the Census categories with the addition of Roma and reported that 92% of cases had self-identified ethnicity recorded in 2018 [22]. Information on ethnic or cultural background is summarised in the demographic section of annual reports, however there has been no additional analysis by ethnicity carried out.

*Computerised Infectious Disease Reporting* (CIDR) is an information system developed by the Health Protection Surveillance Centre to manage the surveillance and control of infectious diseases in Ireland. All medical practitioners in Ireland are required to notify certain infectious diseases to the Medical Officer of Health or Director of Public Health. Notification forms and the recording of ethnicity vary by disease. The notification form for tuberculosis, for example, includes separate ethnic categories for South Asian descent and East/South East Asian descent which are not categories used in the Census [23]. The notification forms for HIV and invasive meningococcal disease (IMD)/bacterial meningitis use the Census categories with the addition of Roma [24, 25]. These forms clarify to the medical practitioner that ethnicity should be self-reported and should not be ‘given’ by the practitioner. An evaluation of the completeness of national HIV surveillance data in 2014 and 2015 reported that ethnicity was recorded for 75.6% of cases in 2014 and 70.7% of cases in 2015 [26]. The recording of country of birth was more complete (89.9% of cases in 2014 and 86.0% of cases in 2015). The annual report for IMD in 2017 reported that ethnicity was known/specified for 32% of cases (28% White, 3% Irish Traveller and 1% Roma) and unknown for 68% of cases [27]. O’Connor et al. reported on a prolonged outbreak of IMD in an extended Irish Traveller family using data from CIDR [28]. Through Traveller health primary care projects, a vaccination awareness initiative was subsequently carried out with a particular focus on health education in meningitis for Irish Travellers [29].

The National Perinatal Epidemiology Centre conducts clinical audits using the *Perinatal Mortality Surveillance System*, the *Severe Maternal Morbidity Audit* system and the *Surveillance of Home Births* database. Ethnicity is recorded for all three using the Census categories. Ethnicity was recorded for all women planning a home birth in 2016 [30]. Women who identified as *Other White* were over-represented in women planning a home birth compared to the general population. Ethnicity was recorded for 98% of women who experienced severe maternal morbidity [31] and 99.2% of perinatal deaths in 2016 [32]. Ethnic minorities were over-represented in both severe maternal morbidity and perinatal deaths, compared to the general population. Evidence, from multiple sources, of higher risks for some ethnic minorities informed the action of the current National Maternity Strategy to ‘*provide additional supports to pregnant women from vulnerable, disadvantaged groups or ethnic minorities*’ [33].

Two disease registers recorded ethnic or cultural background – the *Cystic Fibrosis Registry (CFR)* and the *Irish Childhood Diabetes National Register (ICDNR)*. Cystic Fibrosis is a genetically inherited disease and Ireland has the highest incidence in the world at 7 in every 10,000 people. The most recent annual report of the CFR refers to ethnicity in the context of the calculation of lung function, where it is expressed as a percentage of the expected value from people without cystic fibrosis of the same age, gender, height, and ethnicity [34]. The annual report in 2010 reported that 97.4% of the individuals in the registry identified as the majority ethnic group *White Irish* [35]. The ICDNR is a register for those newly diagnosed with Type 1 diabetes under the age of 15 years. In a review of data from the first six years of the register [36], data collection on ethnicity is referenced as part of the range of socio-demographic data collected on each child but no results by ethnicity are presented.

### National surveys

Five of the ten national surveys in the catalogue recorded ethnic or cultural background.

*Growing up in Ireland* (GUI) National Longitudinal Study of Children is a government funded study which started in 2006 and follows the progress of two groups of children: 8000 nine-year-olds (Child Cohort) and 10,000 nine-month-olds (Infant Cohort). Four waves of data have been collected on both cohorts. GUI records self-identified ethnic or cultural background of the primary caregiver, ethnicity of the child identified by the primary caregiver and self-identified ethnicity of the secondary caregiver using the Census categories. Ethnicity was recorded for 99.3% of primary caregivers in the Infant Cohort with 84% identifying as the majority ethnic group *White Irish*. Variation by ethnic group in breastfeeding rates and introduction of solid foods to infants has been reported using data from GUI [37–39]. Doherty et al. used data from GUI to explore uptake of childhood vaccination and reported on differences by ethnic or cultural background [40]. Cruise et al. reported on the prevalence of maternal depression in the later postpartum period, with ethnic or cultural background used as one of the predictors of prevalence [41].

The *Survey of Lifestyle, Attitudes and Nutrition* (SLAN) was a series of surveys (1998–2007) commissioned by the Department of Health and designed to produce baseline information for the ongoing surveillance of health and lifestyle behaviours in the Irish population. SLAN collected self-identified ethnic or cultural background using the Census categories. Ethnicity was recorded for 98.8% of respondents in 2007 and 90% of respondents identified as ‘White Irish’ [42]. Ethnicity was used, together with other demographic information, to weight analysis to account for non-response and to adjust for socio-demographics in multivariable analysis to predict a range of outcomes, including diabetes [43].

The *Healthy Ireland Survey* is an annual survey of the health and wellbeing of the population commissioned by the Department of Health and is used to support the development and implementation of government policy. It collects information on self-identified ethnic or cultural background using the Census categories [44], however no analysis by ethnicity has been published to date on the first four waves of the survey (2015–18).

The *National Patient Experience Survey* is a nationwide survey asking people for feedback about their stay in hospital, conducted in partnership with HIQA, the HSE and the Department of Health since 2017. It records self-identified ethnic or cultural background using the Census categories. The questionnaire states that this is asked to check if the survey represents all sections of society. The national report for 2018 reported that 97% of respondents indicated an ethnic group and of those that did, the majority (91.7%) indicated having a *White Irish* ethnic background [45]. No additional analysis by ethnicity was reported.

The *European Social Survey* (ESS) is an academically driven, cross-national survey that has been conducted every two years across Europe since 2001. Ireland has participated in all nine rounds of data collection. The questionnaire consists of a core module that is the same in each round and rotating modules that are dedicated to specific topics. The core module includes questions on national identity and ethnicity. Participants are asked if they belong to a minority ethnic group (yes, no), country of birth, parents’ country of birth, citizenship, length of stay if not born in Ireland, and whether they are discriminated against on the basis of the group they belong to, including being a member of an ethnic minority group. The ESS has been used for analysis of ethnic minority health across Europe including an analysis of depressive symptoms among migrants and ethnic minorities [46].

## Discussion

Despite the policy and legal imperatives and the support of an advocacy organisation representing some ethnic minorities, the recording of ethnicity in national health and social care data collections in Ireland is limited with only 14 of the 97 data collections recording information on ethnic or cultural background. Of those that record ethnicity, most used the ethnic categories in the Census which facilitates a standardised approach across data collections and the estimation of rates and ratios using Census population denominators. Roma has been added as a separate group in three of the 14 data collections. This may reflect the advocacy for the collection of ethnic data by the national NGO working to promote rights of members of the Roma community in Ireland. The Central Statistics Office has carried out a public consultation and created an advisory board for the next Census in 2021 with recommendations to change the question asked on ethnicity, including to add Roma as a category.

In line with international best practice, guidance on the collection of ethnicity in all data collections recommended that it should be self-identified by participants/service users and not assigned by staff. Reported rates of identification were generally high (≥90%) with correspondingly low rates of missing data. Rates of ethnicity recording were lower for infectious disease notifications which may reflect that it is not a core variable required for the notification of some diseases [47]. The majority of data collections that recorded ethnicity also recorded at least one other measure related to ethnicity e.g. country of birth. Analysis of outcomes by both ethnicity and country of birth has been recommended to bring new perspectives to understanding health status by accounting for the children of migrants in minority groups or those born abroad who belong to the majority ethnic group [48].

We found no evidence that the integrity of the data collected on ethnicity has been audited across data collections. Data integrity, which measures accuracy and consistency of data, can be assessed by linking data across multiple sources and comparing self-identified ethnicity for the same individual in different data collections or the same individual over time [49]. Data linkage is challenging in an Irish context with a fragmented information technology structure, lack of governance and until recently, no unique health identifier. A new national initiative is underway to develop a proof of concept for infrastructure to support the safe and controlled access, storage, sharing and linkage of routinely collected health and social care data collections [50]. This infrastructure could be used for future exploration of data integrity of ethnicity recording and we identified common identifiers that could be used for data linkage in the Census of Population and five of the eight national data collections that record ethnicity.

National health and social care data collections which recorded ethnicity tended to be focused on potentially high-risk populations e.g. those admitted to psychiatric hospitals, or for treatment for drug or alcohol problems, or with infectious diseases. If the objective of the Racial Equality Directive is to establish a framework to combat discrimination and give effect to the principle of equal treatment for the diverse population of the EU, ethnic data collection limited to high-risk populations is incongruous with that objective. Limiting data collection to these high-risk populations may, in fact, stigmatize members of ethnic minorities and reinforce common myths that migrants are carriers of disease and a burden to the system [51].

There was no routine recording of ethnicity in primary care where the majority of healthcare is delivered or for hospital inpatients, other than psychiatric inpatients. There was also no routine recording of ethnicity in cancer registry or cancer screening data. This contrasts with the UK where incentivisation of ethnicity recording under the Quality and Outcomes Framework improved the completeness of ethnicity data with over 90% of UK general practices recording ethnicity for all of their newly registered patients in 2012 [49] Similarly, high levels of recording of ethnicity (over 80%) have been reported for all acute inpatient and day case records in Scotland from 2015 to 2017 [52]. The National Cancer Registration Dataset in England includes self-reported ethnicity with the completeness of ethnicity data improving over time [53].

There were some examples of where ethnic equality monitoring of the data collections in Ireland, together with the support of advocacy organisations, informed targeted interventions e.g. vaccination awareness initiatives [29] or cultural training for healthcare staff [21]. However, three of the 14 data collections only analysed ethnicity to demonstrate representativeness of the sample to the general population, without any further analysis of outcomes by ethnicity. No published information on ethnicity was available from two other data collections. This points to under-utilisation of existing Irish health information resources to support policy and practice. This is, however, a broader issue in Ireland where lack of capacity for analysis [54] and a fragmented information communication technology structure have been identified as barriers to the use of available databases in general [15].

In an editorial on the collection of data on ethnic origin in the National Health Service in England, Raleigh concluded that ‘*The data are worthless unless they are used to target need and reduce inequalities … the challenge will be for managers, clinicians, commissioners, and providers to use the information to good effect*’ [55]. Without evidence of the use of the data to address health inequities, Varcoe et al. reported that the potential benefits of collecting it in a clinical setting may be outweighed by concerns about the data collection process, particularly for vulnerable patients at risk for discrimination [56]. The importance of collecting other data related to health inequities such as socio-economic status; and accounting for interactions between these variables and ethnicity has also been highlighted [57]. Fulton concluded that successful ethnic monitoring requires a strong regulatory framework complemented by proactive, committed leadership and political will [58]. Creating a culturally-sensitive, equitable health system requires not just underpinning data but a partnership between government and institutions promoting equality and justice, and a strong national health service [59].

This study is part of a larger project which aims to build the evidence base on ethnic minority health in Ireland. The project is planned and governed by an inter-agency Steering Group with community, academic and health sector partners. As part of the project, the implementation of an ethnic identifier in primary care will be researched using an internationally recognised theory of implementation [14]. The results of this study will inform the larger project by providing information for members of the community, healthcare providers and policy makers on where ethnicity is already recorded in the health care system and what it has been used for. The vision for the HSE’s second National Intercultural Health Strategy is a health service that provides high quality, responsive care to all service users from diverse ethnic, cultural and religious backgrounds and empowers these users to access services [12]. The HSE’s goal to build an evidence base on ethnic minority health and ensure evidence-informed practice will be challenging to achieve without more attention to the capacity and technical infrastructure to collect, analyse and continuously report ethnicity data; assessing the quality of the data; and strong leadership and commitment from all stakeholders.

Limitations of this study include using the summary of data content in the national catalogue to search for ethnicity where data dictionaries were not available. Only data collections included in the catalogue were reviewed and data collections under development were not included [15]. Multiple sources (key informants, scoping review, electronic searches and data collection websites) were used to identify examples of where the data on ethnicity had been used but some examples may have been missed.

## Conclusion

This paper reports the first mapping of existing national health and social care data collections with information on ethnicity in Ireland. It identifies that, despite strong policy and legal imperatives, there is limited data collection of ethnicity. While there are some examples of where health differences by ethnicity have been identified and acted upon, a more coordinated and comprehensive approach to the collection, quality and utilization of ethnicity data is needed to promote health equity in Ireland.

## Data Availability

The national catalogue analysed for this study is available as an interactive online catalogue at https://www.hiqa.ie/areas-we-work/health-information/data-collections

## Abbreviations

CFR: Cystic Fibrosis Registry
CIDR: Computerised Infectious Disease Reporting
ESS: Economic Social Survey
EU: European Union
GUI: Growing up in Ireland
HIQA: Health Information and Quality Authority
HSE: Heath Service Executive
ICDNR: Irish Childhood Diabetes National Register
IMD: Invasive meningococcal disease
NDTRS: National Drug Treatment Reporting System
SLAN: Survey of Lifestyle, Attitudes and Nutrition

## Footnotes

1 Irish Travellers are defined as ‘the community of people who are commonly called Travellers and who are identified (both by themselves and others) as people with a shared history, culture and traditions, including historically, a nomadic way of life on the island of Ireland’.
